# Role of Brf1 interaction with ERα, and significance of its overexpression, in human breast cancer

**DOI:** 10.1002/1878-0261.12141

**Published:** 2017-10-27

**Authors:** Zeng Fang, Yunfeng Yi, Ganggang Shi, Songqi Li, Songlin Chen, Ying Lin, Zhi Li, Zhimin He, Wen Li, Shuping Zhong

**Affiliations:** ^1^ Laboratory of General Surgery First Affiliated Hospital Sun Yat‐Sen University Guangzhou China; ^2^ Department of Cardiothoracic Surgery Xiamen University Affiliated Southeast Hospital Zhangzhou China; ^3^ Department of Pharmacology Shantou University Medical College China; ^4^ Cancer Center of Guangzhou Medical University Guangzhou China; ^5^ Department of Biochemistry and Molecular Medicine Keck School of Medicine University of Southern California Los Angele CA USA

**Keywords:** breast cancer, Brf1, ERα, Pol III genes, survival period

## Abstract

TFIIB‐related factor 1 (Brf1) modulates the transcription of RNA Pol III genes (polymerase‐dependent genes). Upregulation of Pol III genes enhances tRNA and 5S RNA production and increases the translational capacity of cells to promote cell transformation and tumor development. However, the significance of Brf1 overexpression in human breast cancer (HBC) remains to be investigated. Here, we investigate whether Brf1 expression is increased in the samples of HBC, and we explore its molecular mechanism and the significance of Brf1 expression in HBC. Two hundred and eighteen samples of HBC were collected to determine Brf1 expression by cytological and molecular biological approaches. We utilized colocalization, coimmunoprecipitation, and chromatin immunoprecipitation methods to explore the interaction of Brf1 with estrogen receptor alpha (ERα). We determined how Brf1 and ERα modulate Pol III genes. The results indicated that Brf1 is overexpressed in most cases of HBC, which is associated with an ER‐positive status. The survival period of the cases with high Brf1 expression is significantly longer than those with low levels of Brf1 after hormone treatment. ERα mediates Brf1 expression. Brf1 and ERα are colocalized in the nucleus. These results indicate an interaction between Brf1 and ERα, which synergistically regulates the transcription of Pol III genes. Inhibition of ERα by its siRNA or tamoxifen reduces cellular levels of Brf1 and Pol III gene expression and decreases the rate of colony formation of breast cancer cells. Together, these studies demonstrate that Brf1 is a good biomarker for the diagnosis and prognosis of HBC. This interaction of Brf1 with ERα and Brf1 itself are potential therapeutic targets for this disease.

**Abbreviations**
Brf1: TFIIB‐related factor 1
DFS: disease‐free survival
ER: estrogen receptor
HBC: human breast cancer
OS: overall survival
Pol III genes: RNA polymerase III‐dependent genes
TFIIIB: transcription factor III B

## 1 Introduction

Breast cancer has become the most common cancer and leading cause of cancer mortality in women in the United States (Siegel *et al*., 2017). Approximately 80% cases of human breast cancers (HBCs) are estrogen receptor positive (ER+), and ~ 20% are ER− (estrogen receptor negative) (Deandrea *et al*., 2008; MacMahon, 2006; Suzuki *et al*., 2008). This finding implies that ERα may play a critical role in breast cancer development. ER+ cases of HBC after hormone treatment by tamoxifen (Tam) have a better prognosis than ER− cases. Tam is currently used for the treatment of both early and advanced ER+ breast cancer in women (Jordan, 1993). Tam is an antagonist of the ER in breast tissue, which competitively binds to the ER, producing a nuclear complex, which leads to a decrease in DNA synthesis and an inhibition of the estrogen effects. Studies have indicated that Tam takes part in the regulation of gene transcription, such as c‐Jun and c‐Fos (Babu *et al*., 2013). Emerging evidence has indicated that alcohol consumption is an established risk factor for breast cancer (Chen *et al*., 2011; Demark‐Wahnefried and Goodwin, 2013; Petr *et al*., 2004; Seitz *et al*., 2012; Singletary and Gapstur, 2001) . The relative increase in risk ranges from 5% to 10% (~ 1 drink/10 g per day) to 40–50% (~ 3 drinks per day) (Singletary *et al*., 1995; Watabiki *et al*., 2000). Alcohol has been classified as carcinogenic to humans by the International Agency for Research on Cancer (Cogliani *et al*., 2011; IARC, 2011; Shi and Zhong, 2017). Therefore, alcohol is also a good reagent to study the mechanism of cell transformation and breast tumor development.

RNA Pol (polymerase) III transcribes a number of noncoding RNA, which include tRNA, 5S rRNA, U6 RNA, 7SL RNA, and 7SK RNA. tRNA and 5S rRNA control the translational and growth capacity of cells (Goodfellow *et al*., 2006; White, 2004). Studies have indicated that oncogenic proteins, such as c‐Myc, c‐Jun, c‐Fos, and Ras, increase Pol III gene transcription (Goodfellow *et al*., 2006; Johnson *et al*., 2008; Zhang *et al*., 2011, 2013; Zhong and Johnson, 2007; Zhong and Johnson, 2009; Zhong *et al*., 2011). In contrast, tumor suppressors, such as BRCA1, p53, PTEN, and pRB, decrease transcription of these genes (Johnson *et al*., 2008; White, 2004; Woiwode *et al*., 2008; Zhong *et al*., 2015). The capacity of these oncogenic proteins and tumor suppressors to alter Pol III gene transcription results from their ability to regulate transcription factor III B (TFIIIB) complex activity. The TFIIIB complex is composed of Brf1, Bdp1, and TATA box‐binding protein (TBP). TBP is an initial and general transcription factor to directly or indirectly regulate RNA Pol I, Pol II, and Pol III gene transcription, whereas Brf1 and Bdp1 specifically regulate transcription of RNA polymerase III‐dependent genes (Pol III genes) (Shi and Zhong, 2017; Zhang *et al*., 2013; Zhong *et al*., 2013a). Our studies have demonstrated that alteration of TBP is able to change the cellular level of Bdp1, but not Brf1 (Zhong and Johnson, 2009), whereas alcohol‐mediated ERα activity affects Brf1, but not TBP (Zhang *et al*., 2013). This finding suggests that Brf1 plays a more important role in the transcription of tRNA and 5S rRNA. Studies have indicated that specific tRNA are upregulated in HBC cells as promoters of breast cancer metastasis (Goodarzi *et al*., 2016). Increased tRNA_i_^Met^ within cancer cells drives cell migration and invasion to enhance the metastatic potential in melanoma (Birch *et al*., 2016). However, the levels of Brf1 expression in human cancers are not well documented. A recent study indicates that Brf1 is overexpressed in hepatocellular carcinoma (Zhong *et al*., 2016). To date, there have been no reports on Brf1 expression in HBC.

Our studies have demonstrated that upregulation of Pol III genes results in increases in cell growth, transformation, and tumor development (Zhang *et al*., 2011, 2013; Zhong and Johnson, 2007, 2009; Zhong *et al*., 2011). The products of the tRNA and 5S rRNA genes are elevated in both transformed cells and tumor cells and biopsies of human cancer, suggesting that they play a crucial role in tumorigenesis (Johnson *et al*., 2008; Woiwode *et al*., 2008; Zhang *et al*., 2011, 2013; Zhong and Johnson, 2007, 2009; Zhong *et al*., 2011). A decrease in Brf1 expression reduces Pol III gene transcription and is sufficient for repressing cell transformation and xenograft tumor formation (Johnson *et al*., 2008; Woiwode *et al*., 2008; Zhong *et al*., 2013a, 2015, 2016). Alcohol increases Brf1 expression to upregulate Pol III gene transcription *in vivo* and *in vitro* (Zhang *et al*., 2013; Zhong *et al*., 2011). Studies have indicated that alcohol administration induces breast tumor formation of alcohol‐fed mice (Wang *et al*., 2012; Wong *et al*., 2012). This finding suggests that alcohol‐caused deregulation of Pol III genes is associated with mammary tumor development. We reported that alcohol increases Brf1 expression through ERα (Zhang *et al*., 2013) and that BRCA1 represses alcohol‐induced Brf1 expression in ER+ breast cancer lines (Zhong *et al*., 2015). However, the levels of Brf1 expression in cases of HBC and the relationship of the levels with a prognosis of HBC patients are unclear.

In this study, we have analyzed 218 cases of HBC. The results indicate that Brf1 is overexpressed in most cases of HBC, which is associated with an ER+ status. HBC cases with high Brf1 expression had a longer survival period than those with lower levels of Brf1. Brf1 and ERα are colocalized in the nucleus, and both interact with each other to mediate Pol III gene transcription. Repression of ERα by the ERα siRNA or tamoxifen decreases the cellular level of Brf1 and reduces the rate of colony formation. These studies show that Brf1 is a new biomarker of HBC diagnosis and prognosis, which will be used as a potential target for HBC therapy.

## 2 Materials and methods

### 2.1 Patients and samples

Paraffin‐embedded tumor tissue samples were obtained from 218 women diagnosed with breast carcinoma who underwent surgical resection between July 2001 and December 2007 in the Department of Breast and Thyroid Surgery and Department of Pathology at the First Affiliated Hospital of Sun Yat‐sen University. We obtained prior patient's consent and approval from the Medical Ethical Committee of the First Affiliated Hospital, Sun Yat‐sen University, for use in these clinical materials in this study.

All patients’ ages ranged from 24 to 79 (median = 50), including 12 cases of *in situ* carcinoma (DCIS), 196 cases of invasive ductal carcinoma (IDC), four cases of invasive lobular carcinoma (ILC), and six cases of metastatic breast cancer (MBC). None of the patients received chemotherapy or radiotherapy before surgery. Clinicopathological information, such as age, tumor size, lymph node status, ER, PR, and HER2 status, was obtained by reviewing medical records and pathology reports.

Fresh tumor specimens were obtained from the patients who underwent resection of the primary breast cancer in the Department of Breast and Thyroid Surgery at the First Affiliated Hospital of Sun Yat‐Sen University. Representative blocks from both the tumor (T) and tumor adjacent noncancerous tissues (N) from each specimen were stored in liquid nitrogen for RNA and protein extraction. Informed consent was obtained from each patient, and the study was approved by the Institute Research Ethics Committee of Sun Yat‐Sen University (ID number: No. [2017]014). None of the patients had previously received chemotherapy or radiation therapy.

### 2.2 Cell line, reagents, and antibodies

The human breast adenocarcinoma cell line MCF‐7 (HTB‐22) was from ATCC (Manassas, VA, USA). Cell culture medium (Dulbecco's modified Eagle's medium [DMEM]/F12), OPTI‐MEM, Lipofectamine 2000, and TRIzol reagent were from Life Technologies (San Diego, CA, USA). Antibodies against ERα (Clone No.33) were from Novus Biologicals (Littleton, CO, USA). Actin mouse monoclonal antibody (2Q1055, Catalog No.SC‐58673) was obtained from Santa Cruz Biotech (Santa Cruz, CA, USA). Mismatch RNA (mm RNA) was described previously (Zhong *et al*., 2013b). The Brf1 antibody (Catalog No.A301‐228A) was from Bethyl laboratories Inc. (Montgomery, TX, USA). The sequences of the primers and Brf1 and ERα siRNA were described previously in Supplements.

### 2.3 Immunohistochemistry

Immunohistochemical staining was performed on formalin‐fixed, paraffin‐embedded sections (4 μm thick) that were deparaffinized in xylene, rehydrated in decreasing concentrations of ethanol, and rinsed in 1× PBS, and then antigen retrieval was performed with microwave treatment in 10 mm EDTA buffer (pH 9.0). Immunohistochemistry (IHC) staining was performed using the EnVision™ Kit (DAKO, Hamburg, Denmark) following the manufacturer's instructions. Endogenous peroxidase activity was quenched by 3% hydrogen peroxide for 15 min. The sections were then incubated with rabbit polyclonal anti‐human BRF1 antibodies (1 : 200) overnight at 4 °C. Next, the tissue sections were sequentially incubated with ready‐to‐use HRP immunoglobulin (EnVision™) for 30 min and then were developed with 3,3′‐diaminobenzidine (DAB) as a chromogen substrate. The nuclei were counterstained with Meyer's hematoxylin.

The levels of Brf1 immunostaining were evaluated independently by two pathologists who were blinded to the survival outcomes of the participants based on the proportion of positively stained tumor cells (stain area) and the intensity of staining. Staining intensities were scored as 0 (no staining), 1 (weak staining) for light yellow color, 2 (moderate staining) for yellow brown color, and 3 (strong staining) for brown color. The positive tumor cell proportion was scored as 0 (no positive tumor cells), 1 (< 5% positive tumor cells), 2 (5–25% positive tumor cells), 3 (25–50% positive tumor cells), and 4 (> 50% positive tumor cells). A modified immunoreactivity score to evaluate the immunostaining results was performed by multiplying the stain intensity by stain area (staining index, SI) as previously described (Li *et al*., 2009). The BRF1 expression levels in breast carcinoma lesions were determined by the SI, which was 0, 1, 2, 3, 4, 6, 9, or 12. An optimal cutoff value was identified as follows: An SI score of > 4 was used to define tumors as high Brf1 expression, and an SI score of ≤ 4 as low.

### 2.4 Western blot analysis

Tissue samples were ground into a powder with liquid nitrogen and lysed in lysis buffer with phosphatase and protease inhibitors. MCF‐7 cells were treated with 25 mm ethanol to extract total cell lysates. Protein concentrations of the resultant lysates were measured by the Bradford method using a Fluostar Omega spectrometer (Cell Biology Core Laboratory of University of Southern California Research Center for Liver Diseases, P30 DK048522). Lysates (50 μg of protein) of tissues or cells were separated by SDS/PAGE and subjected to western blot analysis as previously described (Zhong *et al*., 2011, 2013b). Membranes were probed with specific antibodies against Brf1, ERα, and β‐actin as indicated. A Hybond‐P membrane was used for protein transfer. Bound primary antibody was visualized using horseradish peroxidase‐conjugated secondary antibodies (Vector Laboratories, Burlingame, CA, USA) and enhanced chemiluminescence reagents (Cell Signaling Technology, Danvers, MA, USA). All of the experiments were repeated at least three times.

### 2.5 Immunofluorescence

The collected tumors were fixed with 4% paraformaldehyde, embedded in paraffin, and cut into 4‐μm‐thick sections using a microtome. After removing the paraffin wax with xylene, antigens were retrieved with a microwave treatment in 10 mm EDTA buffer (pH 9.0). The tissue sections were blocked with 5% BSA for 1 h at room temperature and were incubated with rabbit polyclonal anti‐human BRF1 antibodies (1 : 200) or Mouse monoclonal ERα antibody (1 : 100) overnight at 4 °C and then incubated with anti‐rabbit IgG FITC or anti‐rabbit IgG CY3 (Invitrogen Life Technologies Corporation, Invitrogen, Carlsbad, CA, USA) as secondary antibodies (1 : 4000). Nuclear staining of cells was performed using 4,6‐diamidino‐2‐phenylindole (DAPI, Beyotime Biotechnology, Shanghai, China). The slides were mounted in antifade reagent (Invitrogen Life Technologies Corporation). The photomicrographs were captured using an Olympus BX63 fluorescence microscopy (Germany).

### 2.6 Statistical analysis

The ER, PR, and Her2 results in each sample were obtained from the pathology reports. We attempted to categorize the distribution of the ER and PR percentages in two groups according to the description in the methods. For the ER levels, low expression was 0–25% and high expression was ≥ 25%. For the PR levels, low expression was 0–25% and high expression was ≥ 25%. We categorized the positive or negative status of Her2 according to NCCN Guidelines of Breast Cancer.

All statistical analyses were carried out using the spss 22.0 (IBM, Chicago, IL, USA) statistical software package. The chi‐square and Fisher's exact tests were used to analyze the relationship between Brf1 expression and clinicopathological or molecular features. Bivariate correlations between study variables were calculated by Spearman's rank correlation coefficients. Survival curves were plotted by the Kaplan–Meier method and compared using the log‐rank test. A *P* < 0.05 in all cases was considered to be statistically significant.

## 3 Results

### 3.1 Brf1 overexpression and its clinical significance in the cases of human breast cancer

**Figure 1:**
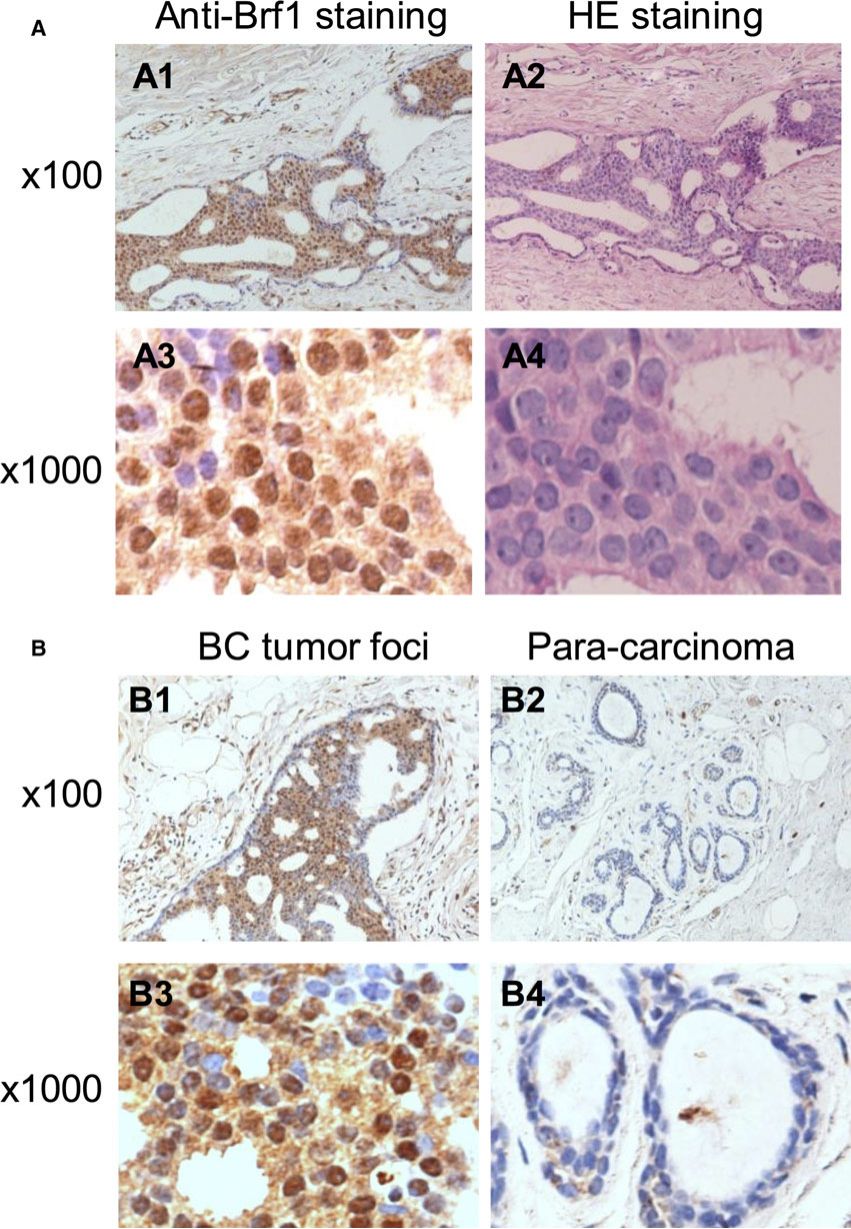
Brf1 IHC staining of samples of HBC. (A); *Brf1 staining*. (A1,A3) IHC staining of Brf1 of HBC tumor tissues; (A2,A4) H&E staining of HBC tumor tissues. (A1,A2) 100× magnification; (A3,A4) 1000× magnification. A representative Brf1 staining of HCC samples. (B); *Comparison of Brf1 staining in tumor foci or para‐can tissue of HBC*. (B1,B3): Strong staining signals of Brf1 expression are seen in tumor foci of HBC; (B2,B4): Weak signals of Brf1 staining are detected in para‐can tissue of HBC. (B1,B3) 100× magnification; (B2,B4) 1000× magnification.

**Figure 2:**
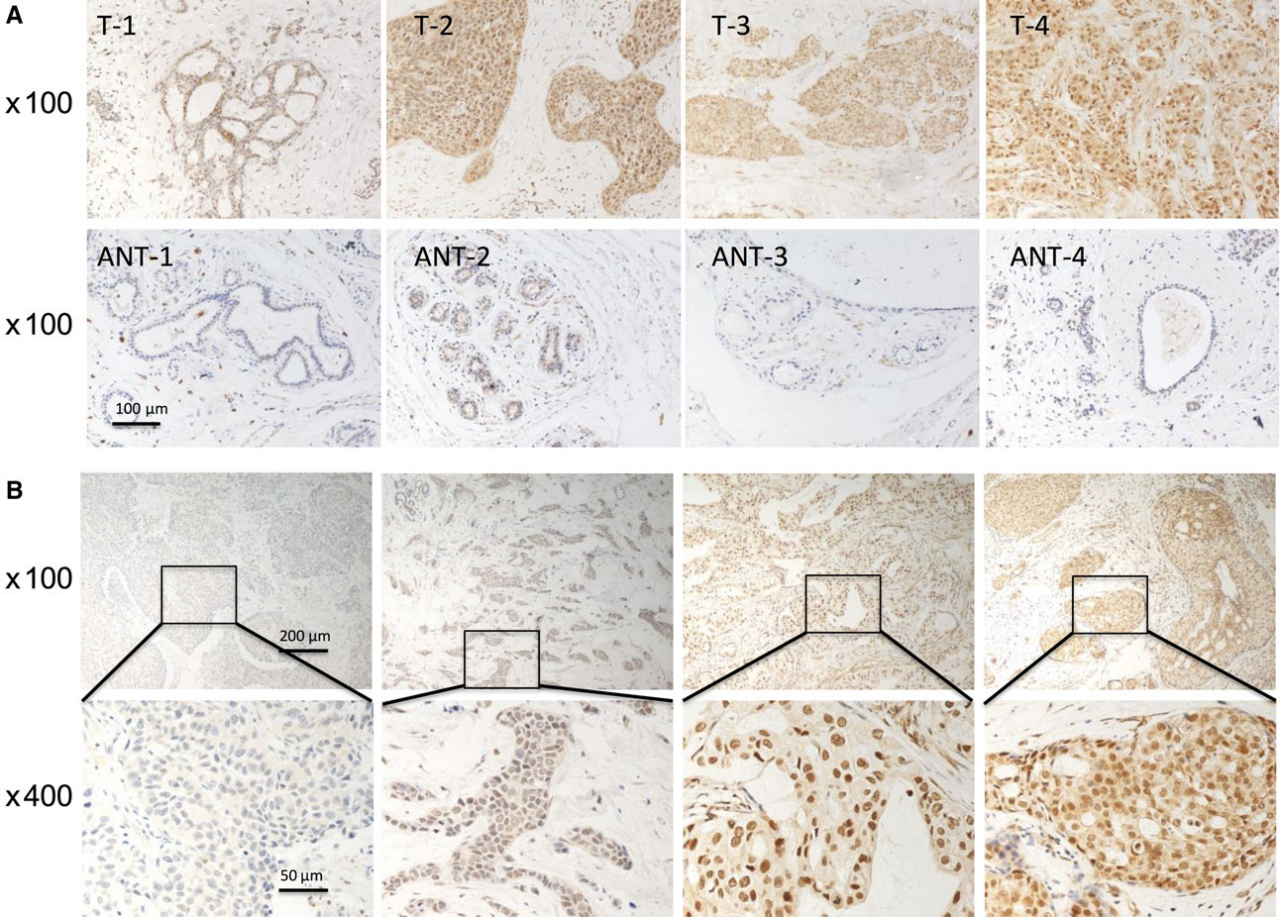
Comparison of Brf1 expression in tumor foci and ANT. (A): *Brf1 staining*. The levels of Brf1 expression were detected in four breast adenocarcinoma lesions (A, upper panel) and their paired ANT (A, lower panel). Brf1 expression was increased in the four breast adenocarcinoma lesions, compared to their matched no cancerous tissues. Magnification, 100×. (B): *Staining intensity of Brf1 in the breast adenocarcinoma tumor tissues*. In terms of the staining intensity of Brf1, the cases were divided into four groups: negative staining, weak staining, moderate staining, and strong staining from left side to right side (B, upper panel). Magnification, up: 100×; down: 400×.

Brf1 is a transcription factor, which specifically regulates Pol III gene transcription. The upregulation of Pol III genes is tightly associated with cell transformation and tumor development. However, Brf1 expression in HBC patients was not determined. To explore the levels and significance of Brf1 expression in the HBC cases, we collected 218 samples from HBC patients and performed IHC analysis using a specific antibody against Brf1. Representative staining results are shown in Fig. 1. Strong Brf1 signals are observed in tumor foci of the HBC tissue compared to the para tissue (around tumor foci; Fig. 1A1,B1). In 218 cases, we defined the four types of staining intensity as negative staining (64/218, 29.4%), weak nuclear staining (50/218, 22.9%), moderate staining (42/218, 19.3%), and strong staining (62/218, 28.4%; Fig. 2B). The difference in Brf1 expression between tumor foci (T; Fig. 2A, upper panels) and adjacent noncancerous tissue (ANT; Fig. 2A, lower panels) is marked. Brf1 primarily accumulates in the nucleus (178/218), in the cytoplasm (8/218), or both (40/218). One hundred and two cases (46.8%) have strong Brf1 staining in lesion tissues with an SI > 4, which is classified as the high Brf1 expression group. The other one hundred and sixteen cases (53.2%) of breast carcinoma include moderate, weak, or negative staining in the lesion tissues with SI ≤ 4, which is classified as low Brf1 expression group. The clinicopathological characteristics of HBC cases are summarized in Table 1 and Table S4 (in supplementary tables). The results indicate that there is not a significant correlation between Brf1 expression and other clinicopathological features, such as patient age, pausimenia, histological type, clinical stage, tumor size, lymph node, and metastasis (Table S4 in supplementary). In contrast, there is a significant correlation between high Brf1 expression and high ER expression (*P* = 0.012), high PR expression (*P* = 0.035), or non‐triple‐negative status (*P* = 0.012), but not Her2 expression (*P* = 0.357; Table 1). These studies indicate that the levels of Brf1 expression of HBC cases are associated with their hormone statuses.

**Table 1:** Correlation between Brf1 expression and molecular features in patients with breast cancer

Molecular features	Patients *n* = 218	High expression (*n* = 102, 46.8%)	Low expression (*n* = 116, 53.2%)	Chi‐squared test *P* value
ER				
High	117	64 (54.7)	53 (45.3)	0.012^a^
Low	101	38 (37.6)	63 (62.4)	
PR				
High	129	68 (52.7)	61 (47.3)	0.035^a^
Low	89	34 (38.2)	55 (61.8)	
HER2				
Positive	66	34 (51.5)	32 (48.5)	0.357
Negative	152	68 (44.6)	84 (55.3)	
Triple‐negative status				
Yes	46	14 (30.4)	32 (69.6)	0.012^a^
No	172	88 (51.2)	84 (48.8)	

a *P *<* *0.05.

### 3.2 Relationship between Brf1 expression and prognosis of breast adenocarcinoma patients

**Figure 3:**
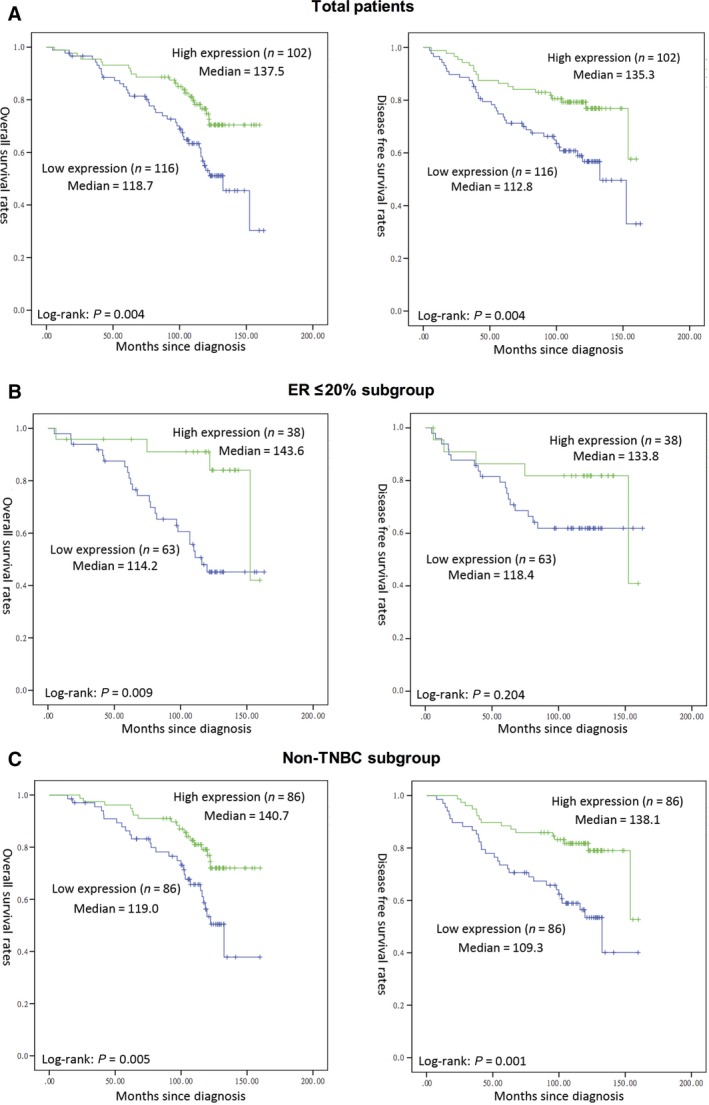
Kaplan–Meier survival curve and log‐rank test analysis of the association between Brf1 expression and HBC patient survival. Brf1 expression of 218 HBC cases was determined by pathological analysis and IHC staining. (A) Total patients; (B) ER low expression or negative subgroup and (C) non‐triple‐negative breast cancer subgroup. *n* = number of patients in the subgroup; *M* = median survival in months of the subgroup. The group of high Brf1 expression or with ER+ status or non‐TNBC group display longer survival period. *P*‐values were calculated by log‐rank test.

We have investigated Brf1 expression levels and the clinical follow‐up information for 218 patients of HBC by Kaplan–Meier analysis and log‐rank test. The results show that the overall survival (OS) times in patients with low Brf1 expression (118.7 ± 5.4 months, *n* = 116) are significantly shorter than one in patients with high Brf1 expression (137.5 ± 4.4 months, *n* = 102, *P* = 0.004; Fig. 3A, left). Furthermore, the disease‐free survival (DFS) months in the high Brf1 expression group (135.3 ± 5.0 months) are markedly longer than those in the low Brf1 expression group (112.8 ± 6.4 months, *P* = 0.004; Fig. 3A, right). These results reveal that patients with high Brf1 expression have better prognosis.

In addition, we have also determined the mean survival times in subgroups of patients with different ER status and triple‐negative status. The results of Kaplan–Meier analyses indicate that the patients in the low Brf1 expression group with low or negative expression of ER have significantly shorter survival times, compared to those in the high Brf1 expression group, *P* = 0.009 (Fig. 3B). There is not a significant difference in the OS or DFS times in the ER high expression subgroup. Similar results, both OS and DFS, are revealed in the cases with high Brf1 expression compared to those with low Brf1 expression in non‐triple‐negative status (*P* = 0.005 or *P* = 0.001; Fig. 3C). However, TNBC patients (46 cases) do not display this kind of OS feature (Fig. S1). The TNBC group (*n* = 16) with high Brf1 expression reveals shorter DFS period (108 months) when comparing to those (*n* = 30) with low Brf1 expression (120 months).

### 3.3 Change in cellular level of ERα alters Brf1 expression

**Figure 4:**
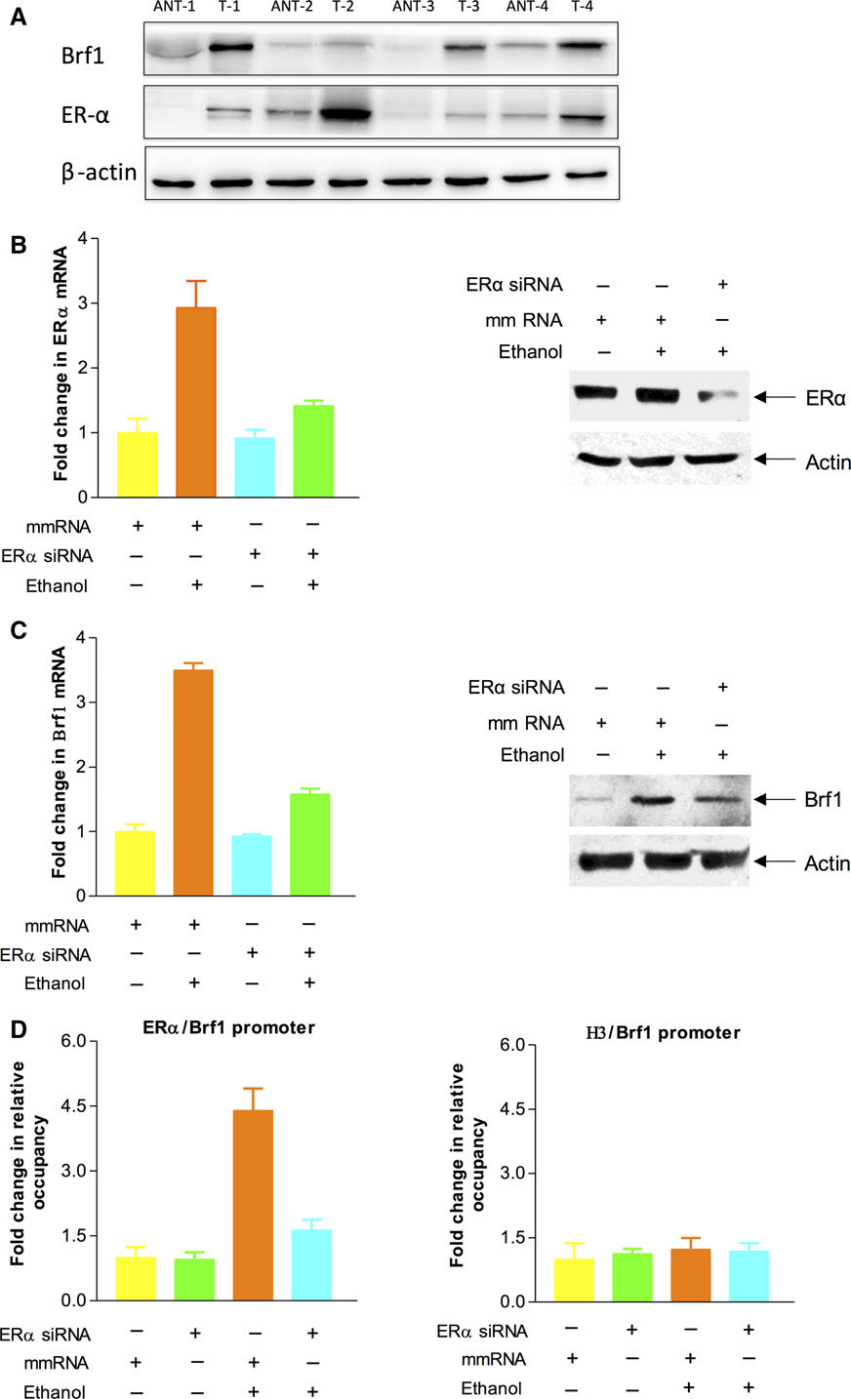
Repression of ERα decreases expression of Brf1. (A): *Expression of Brf1 and ER*α *in breast adenocarcinoma*. Western blots show that the expression levels of Brf1 and ERα protein in four breast adenocarcinoma tumor tissues (T) are markedly higher than those of their paired ANTs. β‐Actin was used as a loading control. (B–C): *ER*α *siRNA decreased the induction of ER*α *and Brf1 caused by ethanol*. MCF‐7 cells were transfected with mismatch RNA (mm RNA) as a control RNA or ERα siRNA for 48 h and treated with ethanol as described previously (Zhang *et al*., 2013). The cell lysates and total RNA were extracted from these cells to determine protein levels of ERα, Brf1, and β‐actin by western blot (B and C, right panels). mRNA levels of ERα and Brf1 were measured by RT‐PCR (B,C, left panels). (D): ERα occupancy of Brf1 promoter: Chromatin was extracted from ethanol‐treated MCF‐7 cells to carry out ChIP assay with ERα or histone H3 antibodies, respectively. H3 is used as a control. These results indicate that ERα modulates Brf1 expression.

To investigate the relationship between ER status and Brf1 expression, we measured the cellular levels of ERα and Brf1 in the biopsies of HBC patients by western blot analysis. The results indicate that the levels of ERα or Brf1 protein in HBC tumor foci are higher than those in ANT (Fig. 4A). High levels of ERα are accompanied by Brf1 overexpression in the samples from the HBC patients (Fig. 4A). This implies that ERα may modulate Brf1 expression. To further explore the mechanism of high Brf1 expression with a longer survival period of ER+ cases, we treated the ER+ breast cancer line MCF7 cells with ethanol, which has been classified as a carcinogen to humans. The results indicate that ethanol causes ~ 3‐fold increase in ERα and Brf1 mRNA levels (Fig. 4B,C, left), but also slightly augments their protein levels (Fig. 4B,C, right). Next, we transfected MCF7 cells with ERα siRNA, compared to the control RNA [mismatch (mm) RNA]. The results show that ERα siRNA reduces ERα expression and decreases cellular levels of Brf1 mRNA and protein (Fig. 4B,C). Furthermore, ethanol increases ERα occupancy of the Brf1 promoter (Fig. 4D). In contrast, ERα siRNA lessens its occupancy on the promoter. These studies demonstrated that ERα indeed modulates Brf1 expression.

### 3.4 Both Brf1 and ERα modulate transcription of RNA Pol III genes

**Figure 5:**
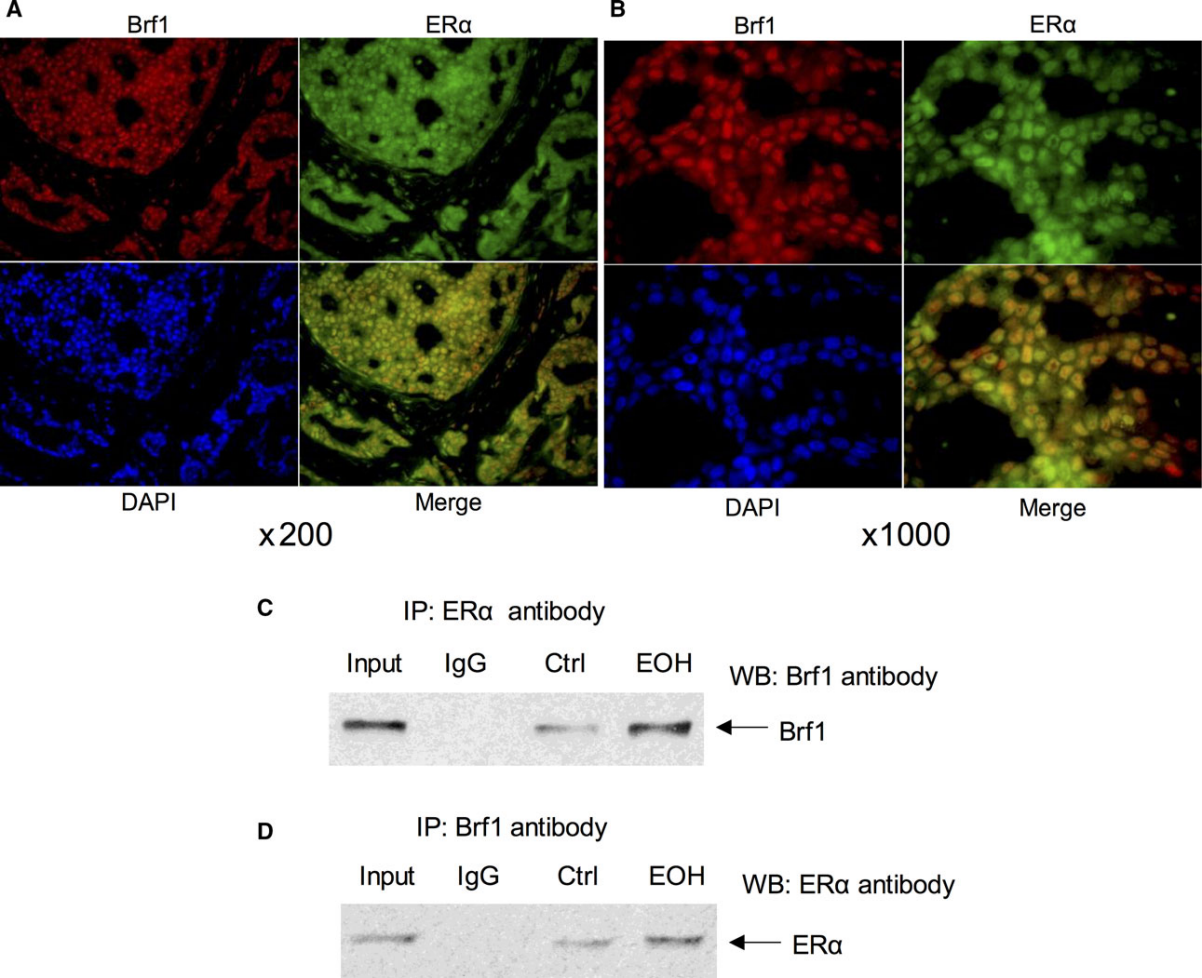
Colocalization and Interaction between Brf1 and ERα. *Colocalization*: Brf1 (red) and ER‐α (green) of the human breast adenocarcinoma tumor tissues were determined by immunofluorescence staining (A). The results indicate that both Brf1 and ERα are localized in nucleus of the tumor cells. Merging picture clearly shows that Brf1 and ERα reveal colocalization in nucleus of HBC biopsy (B). Magnification, up: 200×; down: 1000×. *Interaction between Brf1 and ERα*: MCF‐7 cells were treated with ethanol to extract cell lysates and to perform immunoprecipitation with Brf1 and ERα antibodies, respectively. Western blot analysis indicates that Brf1 antibody is able to put down ERα protein (C), whereas the antibody of ERα can also precipitate Brf1 protein (D). The input samples at C and D were from the ethanol‐treated cells. The results reveal the interaction of Brf1 with ERα in ER+ breast cancer cells.

**Figure 6:**
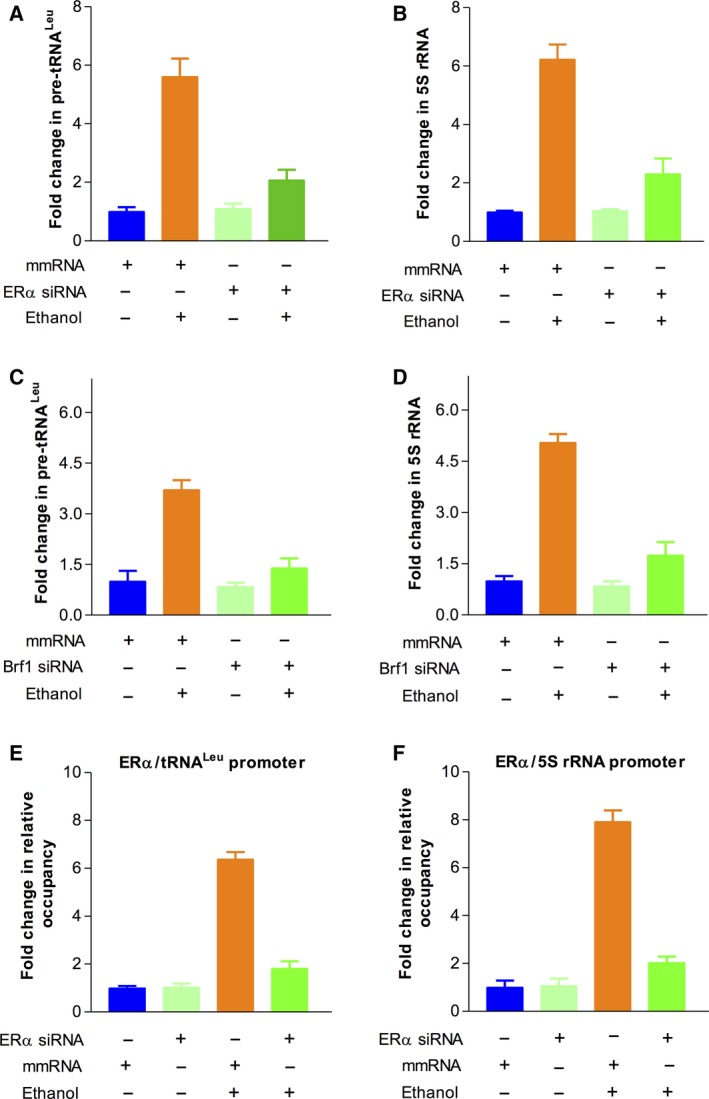
ERα modulates Pol III gene transcription. (A‐D): MCF‐7 cells were transfected with mismatch (mm) RNA, ERα siRNA, or Brf1 siRNA for 48 h, respectively. The cells were treated as described above. The amounts of pre‐tRNA^L^ ^eu^ (A,C) and 5S rRNA (B,D) were measured by RT‐qPCR. Repression of ERα (A‐B) or Brf1 (C–D) by their siRNA decreases Pol III gene transcription. The fold changes are calculated by normalizing to the amount of GAPDH mRNA. (E–F) ChIP assay: MCF‐7 cells were treated as described above in Fig. 4. The results indicate that ERα occupies the promoter of tRNA ^leu^ and 5S rRNA. The bars represent mean ± SE of at least three independent determinations.

To explore whether there is a synergistic role of ERα and Brf1 in Pol III gene transcription, we performed an ERα and Brf1 colocalization analysis by immunofluorescence staining for the HBC tissues. The results show that both ERα and Brf1 have positive staining in ER+ tumor tissues (Fig. 5A). Interestingly, ERα and Brf1 are colocalized in the nuclei of the tumor cells (Fig. 5B). This result displays that ERα and Brf1 may interact to modulate Pol III genes. To investigate this interaction, we performed coimmunoprecipitation assay with ERα and Brf1 antibodies. As we can see, the ERα antibody is able to put down Brf1, whereas Brf1 antibody can also precipitate ERα (Fig. 5C,D). These studies show that the interaction between ERα and Brf1 exists. Further analysis indicates that repression of either ERα or Brf1 by their siRNA decreases tRNA^Leu^ (Fig. 6A,C) and 5S rRNA (Fig. 6B,D) transcription. To identify whether ERα directly modulates Pol III genes, we performed a ChIP assay. The results reveal that ERα occupies the promoters of tRNA^Leu^ and 5S rRNA (Fig. 6E,F). These studies demonstrate that both ERα and Brf1 modulate Pol III gene transcription.

### 3.5 Alteration of Brf1 and Pol III gene transcription causes cell phenotypic change

**Figure 7:**
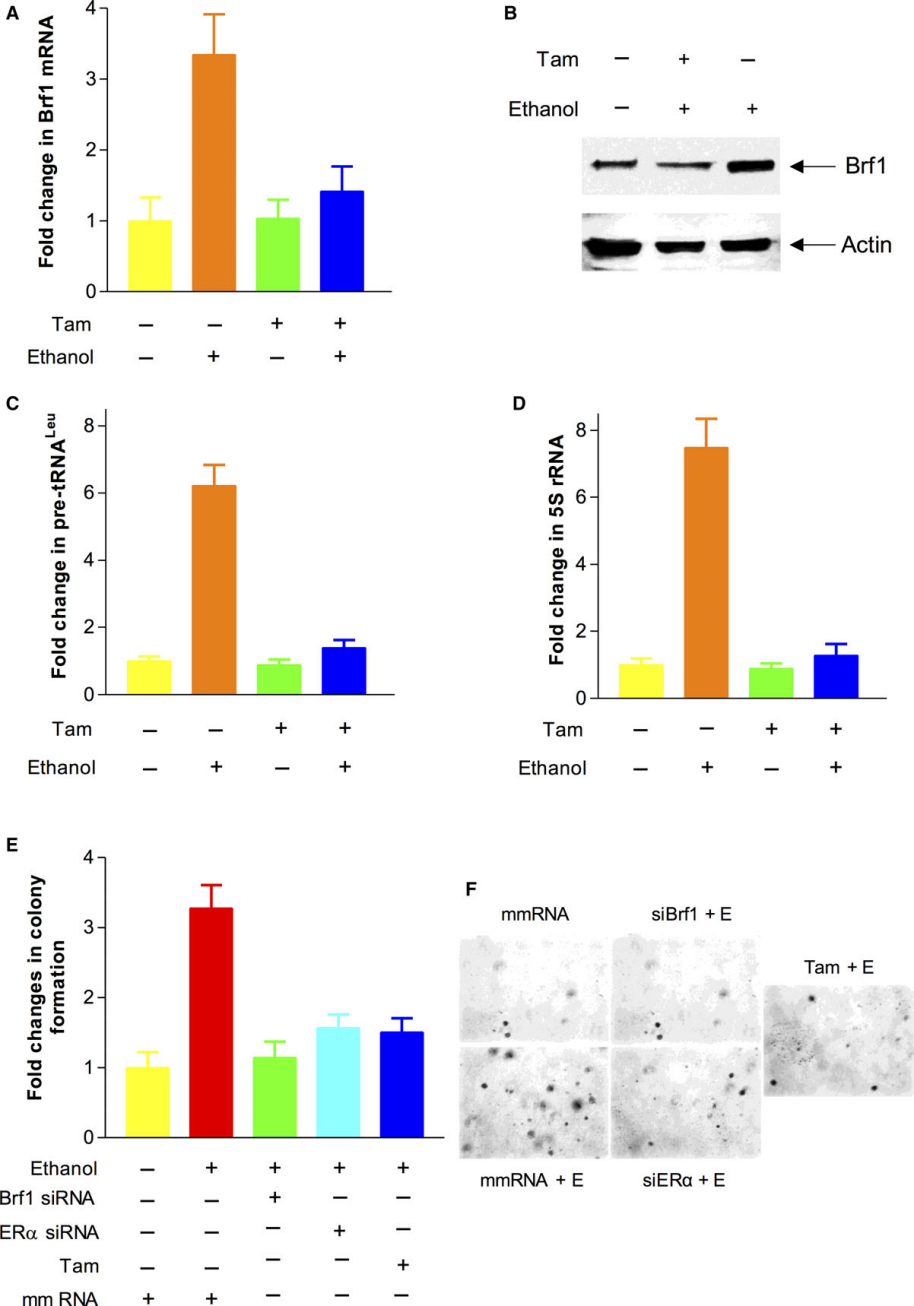
Brf1 and Pol III genes are mediated by Tam. (A–D): MCF‐7 cells were treated with 12.5 μm Tam (tamoxifen). The cells were treated as described above. The levels of Brf1 protein were measured by western blot (B). RT‐qPCR was performed to determine the amounts of Brf1 mRNA (A) and pre‐tRNA^L^ ^eu^ (C) and 5S rRNA transcription (D). The fold changes were calculated by normalizing to the amount of GAPDH mRNA. The bars represent mean ± SE of at least three independent determinations. (E–F): Soft agar assay: MCF‐7 cells were transfected with mm RNA, ERα siRNA, or Brf1 siRNA for 48 h, respectively. The cells were seeded in 6× well plates and treated with ethanol (25 mm) and Tam (12.5 μm) as previously described (Zhong *et al*., 2014). The cells were analyzed for colony formation in soft agar. Colonies were counted at 2–3 weeks after plating. Values are the means ± SE (*n* ≥ 3). *P < 0.05 as indicated.

The above studies have shown that ER+ cases of HBC with high Brf1 expression display better prognosis. Therefore, we have further determined an underlying mechanism of hormone therapy by Tam, which is a good medicine for ER+ cases of HBC. We treated ER+ breast cancer MCF7 cells with Tam and then determined the alteration of Brf1 expression and Pol III gene transcription. The results indicate that Tam decreases cellular levels of Brf1 mRNA and protein (Fig. 7A,B). Tam treatment also reduces transcription of tRNA^Leu^ and 5S rRNA (Fig. 7C,D). Furthermore, we performed a soft agar assay to test whether Tam affects colony formation. The results reveal that Tam treatment decreases the rate of colony formation (Fig. 7E). Thus, these studies show that the effects of Tam on Brf1 and Pol III genes cause phenotypic changes in colony formation.

## 4 Discussion

**Figure 8:**
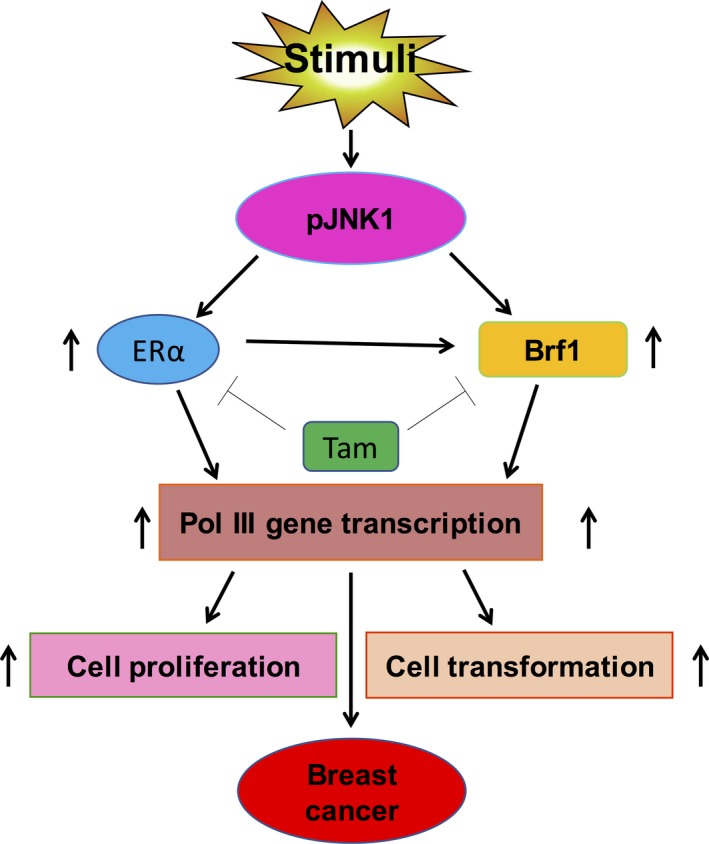
Schematic illustration of Brf1 and ERα mediating Pol III gene transcription. Stimulus induces activation of JNK1 to increase cellular levels of Brf1 and ERα. Tam represses Brf1 expression and reduces ERα activity. The interaction of Brf1 with ERα in turn upregulates Pol III gene transcription to promote cell proliferation and transformation, eventually resulting in breast cancer development.

In the studies, we have presented the mechanistic analysis of ER+ breast cancer and measured the levels of Brf1 expression in 218 cases of HBC for the first time. The results indicate that Brf1 is overexpressed in most HBC cases. Brf1 is mainly localized in the nuclei of HBC tumor cells. The levels of Brf1 in tumor foci are much higher than those of ANT. The cases of HBC with high Brf1 expression have a longer survival period. There is a significantly different survival period in high Brf1 expression with ER+ and PR+ statuses, compared to ER− and PR− statuses. Further analysis indicates that alteration of ERα affects Brf1 expression and Pol III gene transcription. ERα and Brf1 are colocalized in nuclei. The interaction of Brf1 with ERα plays an important role in regulating Pol III gene transcription. Tam inhibits the expression of Brf1 and Pol III genes, resulting in reducing the rate of colony formation (Fig. 8). Brf1 may be used as a new indicator of HBC diagnosis and prognosis. These studies uncover a novel mechanism and the significance of Brf1 overexpression in this disease, which is also a new interpretation of the efficacy on ER+ HBC by Tam treatment.

Overall survival is the period from surgical resection to death, which includes cancer recurrence and metastasis or nature death (noncancer reasons, such as aging and so on); however, DFS is the time from surgical resection to cancer recurrence and metastasis. The patients of DFS mainly died from cancer. In this study, ER+ and PR+ cases with high Brf1 expression represent most part of non‐TNBC patients, who have best outcomes with much late recurrence or metastasis, and longer survival time. For the group with high Brf1 expression, the difference between OS (137.5 months) and DFS (135.3 months) is about 2 months (Fig. 2A, green lines). The group with low Brf1 expression, representing most cases of TNBC, had worse outcomes including earlier cancer recurrence and metastasis. The difference between OS (118.7 months) and DFS (112.8 months) for the group with low Brf1 expression is about 6 months (Fig. 3A, blue lines). Thus, the period (6 months) between OS and DFS of the patients with low Brf1 expression is longer than the time (2 months) for those with high Brf1 expression. The analysis of clinical information demonstrates that ER+ patients with high Brf1 expression display good prognosis.

Although studies on breast cancer have been well documented, to date, there have still been no reports on the mechanism and significance of Brf1 expression in HBC. Alcohol consumption is consistently associated with the risk of breast cancer (Deandrea *et al*., 2008; MacMahon, 2006; Suzuki *et al*., 2008). Alcohol feeding prompted mammary tumor formation (Wang *et al*., 2012; Wong *et al*., 2012). Our studies have demonstrated that alcohol increases Brf1 expression and Pol III gene transcription to facilitate cell transformation and tumor formation (Zhang *et al*., 2013; Zhong *et al*., 2011, 2016). BRCA1 is a tumor suppressor (Duncan *et al*., 1998; Yoshida and Miki, 2004), which is responsible for repairing damaged DNA (Chen *et al*., 2011). Women with an abnormal *BRCA1* gene have up to an 80% higher risk of developing breast cancer (Friendenson, 2007). We determined that restoring BRCA1 in HCC 1937 cells, which is a BRCA1‐deficient line, represses Pol III gene transcription (Zhong *et al*., 2015). More interestingly, overexpression of BRCA1 in MCF‐7 decreases the induction of tRNA^Leu^ and 5S rRNA genes caused by alcohol (Zhong *et al*., 2015). Thus, alcohol is a good reagent to explore the underlying mechanism of breast cancer. As Brf1 specifically regulates tRNA^Leu^ and 5S rRNA gene transcription, it implies that Brf1 may play a critically important role in HBC development. In the present study, the results indicate that ERα modulates Brf1 expression and Pol III gene transcription (Figs 4 and 6). Repression of ERα decreases ethanol‐caused induction of Brf1 and Pol III genes (Figs 4 and 6), whereas human sample studies have shown that Brf1 overexpression is significantly linked to ER+ and PR+ statuses of HBC cases (Figs 1 and 4A, Table 1). Increase in transcription of Brf1 and Pol III genes promotes tumor formation (Johnson *et al*., 2008; Zhong *et al*., 2011), while high Brf1 expression results in shorter survival period for the patients of hepatocellular carcinoma (Zhong *et al*., 2016). However, the HBC cases with low Brf1 show worse prognosis, compared to those with high Brf1 expression (Fig. 3). This is because most of the cases with low Brf1 expression were associated with TNBC (triple‐negative breast cancer), which were more difficult to treat by hormone therapy, resulting in shorter survival period. In contrast, the HBC cases with high Brf1 expression were at ER+ and PR+ status, and hormone therapy was more effective in these cases. Thus, their prognosis is better than those with low Brf1 expression. Here, our results *in vitro* (Figs 4 and 6) further demonstrated that ERα positively modulates Brf1 expression and Pol III gene transcription. This supports an idea that the difference in survival periods between high and low Brf1 expression is dependent on ER+ expression and efficacy of hormone treatment, such as Tam.

In clinical practice, Tam was used to treat ER+ HBC cases. The HBC patients with high Brf1 expression are in the ER+ group. After the treatment, these patients revealed better prognosis. This is because Tam represses Brf1 and Pol III gene expression (Fig. 7). However, TNBC patients (46 cases) did not display this kind of OS feature (Fig. S1). The TNBC group (*n* = 16) with high Brf1 expression revealed shorter DFS period (108 months) when comparing to those (*n* = 30) with low Brf1 expression (120 months). This result further proves that high Brf1 expression is associated with better prognosis in ER+ HBC patients. However, as we did not gain enough TNBC cases samples, the result did not display significance (*P* > 0.05). We will collect additional TNBC case samples to observe this feature. As described above, although this is an unexpected result, it shows the tissue specificity of breast cancer, namely ER+ and PR+. The analysis explains why the cases with low Brf1 expression had worse prognosis.

As ~ 80% of HBC cases are ER+, these cases who received hormone treatment with Tam display a longer survival period and good prognosis (Fig. 3). Tam is widely used in postmenopausal ER+ women with HBC. However, Tam acts as an estrogen agonist, leading to certain adverse effects. Hot flashes are the most common side effect caused by Tam, which affects up to 80% of women. Hot flashes intolerances lead to a severely decreased quality of life and treatment compliance in patients. Endometrial hyperplasia is another common adverse effect in clinical practice, leading to about a 2.5 times higher risk of developing endometrial cancer (Henry *et al*., 2008; Jordan, 2014; Osborne, 1998) . Clinically, cases of Tam resistance are met often. Resistance to endocrine therapies is a major issue in recurrent ER+ HBC patients (De Marchi *et al*., 2016). Several mechanisms have been connected to endocrine resistance, such as mutation in the ligand‐binding domain of the ER (Toy *et al*., 2013), enhanced growth factor signaling, altered DNA methylation of specific genes (Graff *et al*., 1995; Widschwendter and Jones, 2002), or the dysregulation of metabolic pathways (Wang *et al*., 2016). In our study, we discovered a novel mechanism demonstrating that Tam decreases Brf1 expression and Pol III gene transcription (Fig. 7A,B) to inhibit ethanol‐promoted colony formation of breast cancer cells (Fig. 7C). In addition, our study indicates that Brf1 induction is required for ethanol to increase colony formation in soft agar (Fig. 7E), which supports an idea that ethanol‐enhanced Brf1 expression may promote alcohol‐associated breast cancer development. The new finding not only raises our mechanism of understanding the disease, but also provides a possible therapy through repressing Brf1 expression. Thus, developing an inhibitor of Brf1, which is a downstream component of ER pathway, is more important to enhance the efficacy of Tam on the HBC patients by repressing Brf1 expression. TNBC refers to any HBC that does not express the ER, PR, or Her2 genes. This property makes it more difficult to treat TNBC patients, as most hormone therapies target one of the three receptors. More interestingly, our studies indicate that low Brf1 expression is significantly associated with the HBC triple‐negative status (*P* = 0.012; Table 1), which further suggests that Brf1 expression is strongly associated with ER status. Therefore, a decrease in the cellular level of Brf1 is a new direction as therapy of HBC ER+ patients and the patients with Tam‐resistant and/or triple‐negative status.

In summary, we determined that the cases of HBC with high Brf1 expression have a longer survival time and good prognosis. Mechanistic analysis indicates that repression of ERα decreases cellular levels of Brf1 mRNA and protein. The interaction of Brf1 with ERα synergistically regulates Pol III gene transcription. Tam inhibits the expression of Brf1 and Pol III genes and decreases the rate of colony formation, which indicates new implication of the mechanism of Tam treatment for HBC patients. These findings provide a new direction to develop drugs inhibiting Brf1 expression and to increase efficacy of the HBC patients by Tam.

## Author contributions

GS, WL, and SZ involved in conception and design and in the analysis and interpretation of data. ZF, YY, and SC involved in data acquisition and in writing and reviewing the manuscript. SL, YL, and ZL involved in the development of the methodology. ZF, YY, GS, and ZH provided administrative, technical, or material support. SZ and WL supervised the study. All authors read and approved the final manuscript.

## Supporting information


**Fig. S1**. Kaplan‐Meier survival curve and log‐rank test analysis of the association between Brf1 expression and THBC patient survival.
