# Supplementary material for: Role of Brf1 interaction with ERα, and significance of its overexpression, in human breast cancer
Source: Mol Oncol. 2017 Oct 27;11(12):1752–67. doi: 10.1002/1878-0261.12141 (PMC5709663; doi:10.1002/1878-0261.12141)
Supplement: Supplementary file 1 — Fig. S1. Kaplan‐Meier survival curve and log‐rank test analysis of the association between Brf1 expression and THBC patient survival. [file MOL2-11-1752-s001.pdf]

Supplements

Chromatin immunoprecipitation (ChIP) assays.

MCF**-7** cells (3 x 10^6 cells) were cultured in 15 cm dishes and treated with ethanol. The cells were fixed with formaldehyde (1% final concentration) at 24°C for 10 min. Soluble chromatin were prepared as described previously (5). The chromatin were then diluted 1:10 with buffer (0.01% SDS, 1.1% Triton X-100, 1.2 mM EDTA, 16.7 mM Tris−HCl and 167 mM NaCl) and were subjected to immunoprecipitation (IP) in lysis buffer (50 mM Tris−HCl, 10 mM EDTA, 1% SDS) and a protease inhibitor cocktail set (CalBiochem). Pre-immune serum was used as a control and antibodies of Brf1, ERα or histone H3 were used for IP. The chromatin and antibodies were incubated at 4°C overnight. Complex of chromatin/antibody were recovered by adding 45 μl of protein A/G PLUS−agarose beads and incubated at 4°C for 2 h. The beads were sequentially washed for 10 min each in 1 ml of low salt, high salt and LiCl immune complex wash buffer. Immunocomplexes were eluted off the beads by incubation with 200 μl of 1% SDS and 50 mM $NaHCO_3$. The eluents were incubated at 65°C for 6 h to reverse the formaldehyde-induced protein−DNA crosslinks. Extracted DNAs were resuspended in 100 μl of TE and qPCR were performed for amplification (5). The primer sequences that were used are shown in **Table S3** in Supplementary Data. The fold change in promoter occupancy was calculated by setting the level of promoter occupancy in the cells without ethanol treatment at 1.

Real time quantitative PCR (RT-qPCR) and transfection

Liver cancer cell lines and non-tumor liver cell lines were grown to 85% confluence and starved in serum-free for 3-4 h. The cells were treated with 50 mM ethanol for another 1 h. Total RNAs of the cells and the liver tissues from human and mice were extracted with TRIzol reagent (Invitrogen). For siRNA transfection assays, AML-12 cells were cultured in 10% FBS/DMEM-F12 medium as described previously [17]. Serum-free medium was added to each dish with Lipofectamine-2000 Brf1 siRNA or mmRNA complexes, and cells were further incubated for 4 h at 37◦C. The cells were incubated for 48 h before harvesting. Total RNA samples were quantified and reverse-transcribed. After first-strand cDNA synthesis, the real time qPCR (RT-qPCR) were performed with specific primers (**Supplements, Table S4**) and PCR reagent kits (Bio-Rad Biotech) in the ABI prism 7700 Sequence Detection System. Precursor of $tRNA^{Leu}$ and 5S rRNA transcripts and Brf1 mRNA were measured by RT-qPCR as described previously [17].

Cell anchorage-independent growth

AML-12 cells (1 x 10^4 cells/well in 6-well plate) were transfected with mismatch RNA (mmRNA) or Brf1 siRNA (**Supplements, Table S3**). The cells were suspended in 0.35% (w/v) agar in 10% FBS/DMEM/F12 with or without 50 mM ethanol, 20ng/ml EGF or both EGF and ethanol over a bottom layer of media with 0.5% (w/v) agar. Cells were fed fresh complete media with EGF or/and ethanol twice weekly. Colonies were counted 3-4 weeks or longer after plating as previously described [16].

Table S1. Primer Sets for Quantitative RT-PCR

**Target**	**Primers**	**Annealing Temperature**
Pre-tRNA^{Leu} (1)	(F) 5'-GTC AGG ATG GCC GAG TGG TCT AAG-3'<br>(R) 5'-CCA CGC CTC CAT ACG GAG AAC CAG AAG ACC C-3'	61º
5S rRNA (2)	(F) 5′ GGC CAT ACC ACC CTG AAC GC 3′<br>(R) 5′ CAG CAC CCG GTA TTC CCA GG 3′	61º
Human Brf1 (3)	(F) 5’ CCT CGG GCC TCT GCG GAG CAG -3’<br>(R) 5’ TCA TCA ATG GTC AAC TGA CTG GTG G -3’	60º
GAPDH (2)	(F) 5′-TCC ACC ACC CTG TTG CTG TA-3′<br>(R) 5′-ACC ACA GTC CAT GCC ATC AC-3′	61º

Abbreviations: (F) = forward, (R) = reverse.

Table S2 Primer Sets for ChIP

**Target**	**Primers**	**Annealing Temperature**
hBrf1 promoter –233/+42 (3)	(F) 5’ CGT CCA GCT TTA GTC CCC GAC 3'<br>(R) 5' CGA GCC CAA GGC GGC TTC G 3'	63º
hBrf1 promoter –1153/–941	(F) 5’ GAG ACA GAG TTT CAC TAT TGT C 3’<br>(R) 5’ GGT ACA ACC GGT CCG ACC AGA GC 3’	62º
tRNA^{Leu} gene (1)	(F) 5'-GTC AGG ATG GCC GAG TGG TCT AAG-3'<br>(R) 5'-CCA CGC CTC CAT ACG GAG AAC CAG AAG ACC C-3'	61º
5S rRNA (2)	(F) 5′ GGC CAT ACC ACC CTG AAC GC 3′<br>(R) 5′ CAG CAC CCG GTA TTC CCA GG 3′	61º

Table S3. siRNA targets

Targets	Sequences
Mismatch	sense 5'UUC UCC GAA CGU GUC ACG U 3'<br>antisense 5'ACG UGA CAC GUU CGG AGA A 3'
Human Brf1 siRNA	**A:** sense 5′ GGA AGA UCU GUU GUU ACU U 3′ (4)<br>antisense 5' AAG UAA CAA CAG AUC UUC C 3'<br>**B**: sense 5′ CCC GUG CCU GUA UAU UCC A 3' (4)<br>antisense 5' UGG AAU AUA CAG GCA CGG G 3'<br>**C:** sense 5' GAG CAU AGC GCC AGU GCC A 3'<br>antisense 5' UGG CAC UGG CGC UAU GCU C 3'
Human ERα siRNA:	**A:** sense 5' GGC CAA AUU CAG AUA AUC G 3' (5)<br>antisense 5' CGA UUA UCU GAA UUU GGC C 3<br>**B:** sense 5' AAU GAU GAA AGG UGG GAU A 3'<br>antisense 5' UUA CUA CUU UCC ACC CUA U 3'<br>**C**: sense 5' UGA UGA AUC UGC AGG GAG A 3'<br>antisense 5' ACU ACU UAG ACG UCC CUC U 3'

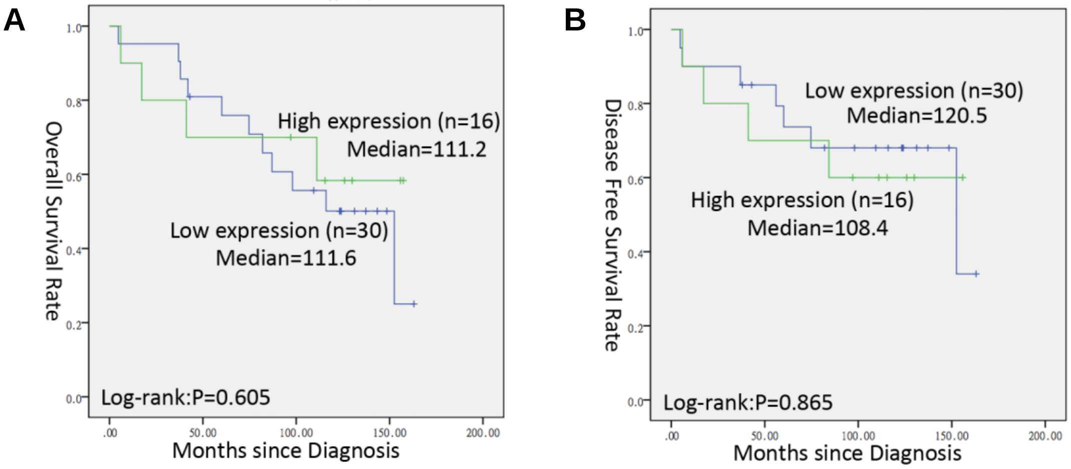

Fig. S1. Kaplan-Meier survival curve and log-rank test analysis of the association between Brf1 expression and THBC patient survival. Brf1 expression of 46 TNBC (triple-negative breast cancer) cases was determined by pathological analysis and immunohistochemistry staining. (**A**) OS (overall survival) period of TNBC patients; (**B**) DFS (disease free survival) period of TNBC cases. n = number of patients in the subgroup, M = median survival in months of the subgroup. The patients with high Brf1 expression in TNBC group display shorter survival period. P-values were calculated by log-rank test.

Table S4 Correlation of Brf1 expression and Clinicopathologic Features in Breast Cancer patients

Clinicopathologic Features	Patients n=218	High expression (n=102, 46.8%)	Low expression (n=116, 53.2%)	X^2 Test *p* value
Age (Year)				0.261
<50 yr	105	53(50.5)	52(49.5)	
≥50 yr	113	49(43.4)	64(56.6)	
Pausimenia				0.647
Yes	99	48(48.5)	51(51.5)	
No	119	54(45.4)	65(54.6)	
Histological type[1]				0.441
DCIS	15	5(33.3)	10(66.7)	
IDC	198	95(48.0)	103(52.0)	
ILC & MBC	6	2(33.3)	4(66.7)	
Clinical Stage[2]				0.925
I	42	20(47.6)	22(52.4)	
II	139	64(46.0)	75(54.0)	
III	37	16(43.2)	21(56.8)	
Tumor size				0.280
≤ 2cm	67	35(67.2)	32(32.8)	
2-5cm	113	47(41.6)	66(58.4)	
> 5cm	38	20(52.6)	18(47.4)	
Lymph nodes				0.355
Positive	116	63(54.3)	53(45.7)	
Negative	102	49(48.0)	53(52.0)	
Distant Metastasis				0.322
Yes	6	4(66.7)	2(33.3)	
No	212	98(46.2)	114(53.8)	

1.DCIS: Ductal carcinoma in situ; IDC: Invasive duct carcinoma; ILC: Invasive lobular carcinoma;

2. Clinical stage accords to the AJCC Tumor-Node-Metastasis staging system.

*p<0.05

Supplemental references

1. Crighton D, *et al*., p53 represses RNA polymerase III transcription by targeting TBP and inhibiting promoter occupancy by TFIIIB. *EMBO J.* 2003; 22: 2810–2820.
2. Winter AG. *et al*., RNA polymerase III transcription factor TFIIIC2 is overexpressed in ovarian tumors. *Proc. Natl Acad Sci USA* 2000; 97: 12619-12624.
3. Zhang Q, Jin J, Zhong Q, Yu XL, Levy D, Zhong S. ERα mediates alcohol-induced deregulation of Pol III genes in breast cancer cells. *Carcinogenesis* 2013; 34: 28-37.
4. Marshall L, *et al*. (2008) Elevated tRNA(iMet) synthesis can drive cell proliferation and oncogenic transformation. *Cell* 133, 78-89.
5. Leivonen SK, *et al*. (2009) Protein lysate microarray analysis to identify microRNAs regulating estrogen receptor signaling in breast cancer cell lines. *Oncogene.* 28:3926-3936.
